# Gram-Negative Rods on Inanimate Surfaces of Selected Hospital Facilities and Their Nosocomial Significance

**DOI:** 10.3390/ijerph19106039

**Published:** 2022-05-16

**Authors:** Ondrej Zahornacký, Štefan Porubčin, Alena Rovňáková, Pavol Jarčuška

**Affiliations:** Department of Infectology and Travel Medicine, Faculty of Medicine, Louis Pasteur University Hospital, Pavol Jozef Šafarik University, 041 90 Košice, Slovakia; ondrejzahornacky@gmail.com (O.Z.); stefan.porubcin@gmail.com (Š.P.); alenarovnakova@gmail.com (A.R.)

**Keywords:** nosocomial, sticks, gram-negative, inanimate, surfaces

## Abstract

Inanimate surfaces are often referred to as nosocomial bacterial reservoirs and represent an important vector in the process of spreading pathogens to patients. Most gram-negative rods can survive on inanimate surfaces for several months. The aim of this study is to determine the prevalence and resistance of gram-negative bacteria isolated from the inanimate surfaces of two selected hospital departments. MALDI-TOF identified gram-negative rods isolated from inanimate surfaces. Antibiotic resistance was determined using a disk diffusion method, and the phenotype of resistance was determined using an inhibitory analyzer. From the inanimate surfaces, 98 strains of gram-negative nosocomial bacteria were identified by the MALDI-TOF MS. The most frequently isolated bacterium occurring in both departments was *Pseudomonas aeruginosa* (*n* = 33), followed by *Acinetobacter baumannii* (*n* = 20) and *Enterobacter cloacae* (*n* = 14). The most common phenotypic type of resistance in both departments was ampicillin resistance—AmpC (*n* = 38), then production of extended-spectrum β-lactamase (ESBL) (*n* = 33), followed by SHV-1 (*n* = 11), TEM-1 (*n* = 11), and fluoroquinolone resistance—Qnr (*n* = 22). The nosocomial important enzymes capable of hydrolyzing carbapenems, OXA-48 and metallo-β-lactamases, were confirmed in 12 and 2 cases, respectively. The results of our study prove that inanimate surfaces in hospitals are a reservoir of resistant gram-negative bacteria, which directly threaten hospitalized patients.

## 1. Introduction

Nosocomial infections are still a current and often discussed topic. Resistance to nosocomial pathogens is constantly growing, and nosocomial infections directly threaten every hospitalized patient, forcing us to delve deeper into this global problem [1,2]. The burden of nosocomial infections is already substantial in developed countries. The incidence in regular wards is from 5% to 15% of hospitalized patients and about 50% or more in intensive care units (ICUs) [3]. Antibiotics play a significant role in the growth in resistance of nosocomial isolates. Reckless and often unindicated administration of antibiotics in the hospital environment has led to multi-resistant hospital isolates. It is estimated that the impact of antibiotic resistance by the year 2050, will result in up to 10 million deaths each year, which exceeds the number of deaths caused by cancer [4].

Infections caused by nosocomial bacteria are often the cause of death, especially in polymorbid patients. In addition, the treatment of nosocomial infections is often costly and leads to the development of further complications (decompensation of chronic diseases, e.g., diabetes mellitus, chronic heart failure, chronic kidney disease, etc.), which prolongs the overall length of hospitalization [5]. Prolonged hospitalization represents an economic burden for the health care facility because it increases the cost of treatment through increased drug consumption, patient isolation, or the need for additional diagnostic and therapeutic interventions [6].

The most common nosocomial infections are catheter-associated urinary tract infections, surgical site infections, central line-associated bloodstream infections, ventilator-associated pneumonia, and *Clostridium difficile* infections [7]. 

The basic premise of nosocomial infections is the presence of the source of infection from which the nosocomial agent spreads. The most common source of nosocomial infections is a hospitalized patient, who is a source of endogenous and exogenous infection. With an exogenous source, the patient produces microorganisms into the surrounding environment, which can be transmitted to other hospitalized patients, medical staff, and visitors to medical facilities (directly or indirectly). Conversely, if the patient’s own microflora becomes the infection’s source, it is an endogenous infection. Another significant source of nosocomial infection is the staff of medical facilities. Spreading the infection through the hands of inanimate surfaces is an equally important aspect. The last potential source of infection is visitors to medical facilities, who come into contact with patients and staff [8,9].

Inanimate surfaces in the hospital environment are among the most critical determinants in the emergence and spread of nosocomial infections. Nevertheless, they are a source of infection in only one-third of cases [10]. Contamination of equipment and high-touch surfaces surrounding the patient by nosocomial bacteria is very high. It is important to note that direct contact with environmental surfaces or equipment transmits most nosocomial infections [11]. Therefore, colonization of these areas by hospital bacteria plays a central role in transmitting the infection to patients. Major nosocomial pathogens have been identified on inanimate surfaces, and they can circulate between hospitalized patients and might persist and survive in the environment for a long time [12,13]. Therefore, it is desirable and essential to know what types of bacteria colonize the inanimate surfaces of hospital facilities and how resistant they are to antibiotics [14].

Nosocomial gram-negative rods are often involved in the etiopathogenesis of nosocomial infections. The most common nosocomial pathogens include *Acinetobacter baumannii*, *Pseudomonas aeruginosa*, *Escherichia coli*, *Klebsiella pneumoniae*, *Enterobacter* spp., *Proteus* spp., *Morganella* spp., *Providentia* spp., and *Serratia* spp. They are the most common cause of urogenital tract infections and pneumonia, and cause various intra-abdominal infections and catheter sepsis [15]. The incidence of ESBL-producing *Enterobacterales* has grown enormously in healthcare facilities over the last two decades. One traditional treatment for ESBL-producing enterobacterial infections has been the use of carbapenems (imipenem, meropenem, doripenem, and ertapenem). However, since 2000, strains of *Enterobacterales* that resist the effects of carbapenems have begun to appear. Carbapenem-resistant *Enterobacterales* (CREs) are spreading to other parts of the world due to antibiotic selection pressure and are endangering hospitalized patients. Mortality from CRE infections ranges from 40% to 50% [16].

It is essential and clear that monitoring the hospital’s inanimate surfaces is vital in controlling nosocomial infections. As possible causes of infection, contamination of surfaces may be mentioned, even if cross-contamination by hands is probably the most significant risk. In addition, hospital inanimate surfaces colonized by different microorganisms constitute unique ecological niches that require cumbersome, complex, and costly procedures necessary for better patient safety [17].

This study aimed to determine the incidence and resistance of gram-negative rods occurring on the inanimate surfaces of two selected departments (Department of Internal Medicine (DIM) and the Department of Anaesthesiology and Intensive Care (DAIC)) of the University Hospital in eastern Slovakia. The reason for choosing this medical facility is that it is the largest regional hospital in eastern Slovakia. According to our observation, the study results reflect the global epidemiological situation associated with the occurrence of gram-negative rods on inanimate surfaces in hospitals in Central and Eastern Europe. Furthermore, the incidence of gram-negative rods on inanimate surfaces at DIM is comparable and correlates with the incidence of these pathogens in long-term care wards; in the case of DAIC, the incidence is comparable and correlates with the incidence in intensive care units (ICUs). Another reason for the selection was that both wards reported the highest incidence of nosocomial infections among all hospital wards and administered a wide range of antibiotics. Therefore, we assumed that the highest incidence of gram-negative nosocomial rods would be on their inanimate surfaces.

## 2. Materials and Methods

### 2.1. Sampling and Transport of Samples to the Laboratory

The research took place at two selected departments—DIM and DAIC, and samples were taken in February 2022. Overall, 82 swabs were collected from DIM and DAIC using sterile collection kits. The most frequent collection points were floors, personal tables, bed handles, X-ray and ECG devices, door handles, infusion pumps, fans, etc. After taking swabs from various inanimate surfaces, the samples were placed in appropriate sterile containers, labelled, and quickly transported to the microbiology laboratory.

### 2.2. Cultivation and Evaluation of Bacterial Growth on Culture Medium

In the laboratory, all collected swabs were inoculated on sterile culture media (blood agar containing 5% ram erythrocytes) and cultured in a thermostat at a constant temperature of 37 °C under aerobic conditions for a total of 24–48 h. After cultivation, the growth of bacteria on individual blood agar was evaluated for each swab separately—absent bacterial colonies (agars remained sterile, POS), 1 bacterial colony—pure bacterial culture, 2 or more bacterial colonies—mixed bacterial culture. The mixed bacterial cultures were then re-inoculated onto additional sterile blood agars and re-cultured under the same laboratory conditions. The aim was to isolate and obtain pure bacterial cultures.

### 2.3. Identification of Samples by MALDI-TOF MS

A MALDI-TOF MS identified pure bacterial cultures. The preparation, identification of the sample, and subsequent evaluation always followed the exact procedure of the instrument manufacturer. The sample preparation method was performed according to the German manufacturer Bruker Daltonics. The determined bacterial colony was applied to the carrier plate of the target instrument using a sterile bacteriological loop and allowed to dry at room temperature for 5–10 min. Each sample was applied in duplicate to increase the success of the identification. After drying, we added 1 μL of the matrix (cinnamic acid). It was allowed to dry again at room temperature for 5–10 min. The target with the prepared bacterial samples was placed in a MALDI-TOF MS instrument. After successful calibration, identifying bacteria using the MALDI Biotyper 3.0 software system was started.

The analysis results are the MALDI-TOF mass spectrum, a protein-peptide profile showing the number of bacterial proteome particles. The identification of microorganisms was performed by comparing the obtained spectrum with a database of known characteristic spectra utilizing the software. In the spectrum analysis, first generic and then species characteristic signals are sought. The software aims to determine the unknown species of the microorganism by calculating the similarity of the mass spectrometer profile and comparing the unknown profiles with the strains recorded in the reference library. The library contains spectra of individual types of microorganisms [18].

### 2.4. Testing of Susceptibility of Identified Bacteria to Selected Antibiotics

Nosocomial gram-negative rods (*n* = 98, 26%) were selected from the group of successfully identified bacteria (*n* = 377) and tested for susceptibility to antibiotics (R, resistance in mm): ampicillin (R < 14), amoxicillin + clavulanic acid (R < 19), cefuroxime (R < 19), trimethoprim + sulfamethoxazole (R < 11), doxycycline (R < 17), cefpodoxime (R < 21), cefoxitin (R < 19), cefotaxime (R < 17), ticarcillin (R < 20), cefepime (R < 24), aztreonam (R < 21), ciprofloxacin (R < 22), imipenem (R < 19), imipenem + EDTA (R < 22), meropenem (R < 16), cefoperazone + sulbactam (R < 22), gentamicin (R < 17), amikacin (R < 18).

Successfully identified, pure, 24 h bacterial cultures, sterile Müller-Hinton agar petri dishes, and antibiotic test kits were used to determine antibiotic susceptibility. Qualitative methods were used-disk diffusion tests. Saline suspensions (turbidity intensity 0.5 McFarland) were prepared from pure bacterial cultures. The suspension was then inoculated onto a Müller-Hinton agar plate. After drying, a dispenser placed antibiotic disks with specific antibiotics on the plate. The plates thus prepared were placed in a thermostat at a constant temperature of 37 °C and cultured for 18–24 h. After culturing, the susceptibility of the test strain to selected antibiotics was assessed by measuring the width of the growth inhibition zone (in mm). The analyzer of the inhibition disk diffusion zone (Bacmed) was used to measure the inhibitory growth zone and determine the degree of resistance.

The determination of the resistance of the isolated bacterial strain and the measurement of the inhibition zone using the Bacmed instrument were conducted according to EUCAST breakpoints [19].

### 2.5. Statistical Analysis

Data entry and analysis were performed using program Gretl version 1.9.5. *p*-value was considered statistically significant when *p* < 0.05. Pearson’s chi-square test was used as a statistical method for data processing and to determine the difference between two examined files within one monitored characteristic.

## 3. Results

**Figure 1:**
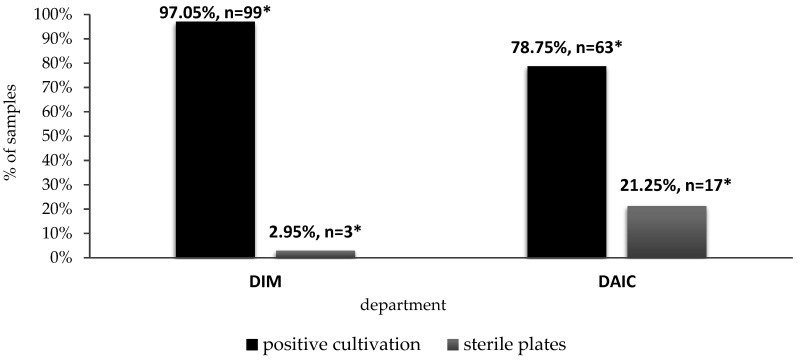
Comparison of the number of sterile plates and positive cultivations on both wards (statistically evaluated by chi-square test) DIM, *n* = 102; DAIC, *n* = 80, *p* = 0.001 (*—significant).

A total of 182 smears (DIM-102, DAIC-80) were taken from both departments. Positive cultivation was recorded in 162 cases (on 162 Petri dishes or broths). In 20 cases, the soils remained sterile after inoculation (DIM, *n* = 3; DAIC, *n* = 17). The cultivation results in both departments are shown in Figure 1. At the DIM and DAIC, positive culture was recorded on 99 and 63 Petri dishes (97.05%; 78.75%), respectively.

Overall, 382 pure bacterial colonies were isolated. All were subsequently subjected to identification on a MALDI-TOF MS, and 377 bacterial cultures (98.7%) were successfully identified.

**Figure 2:**
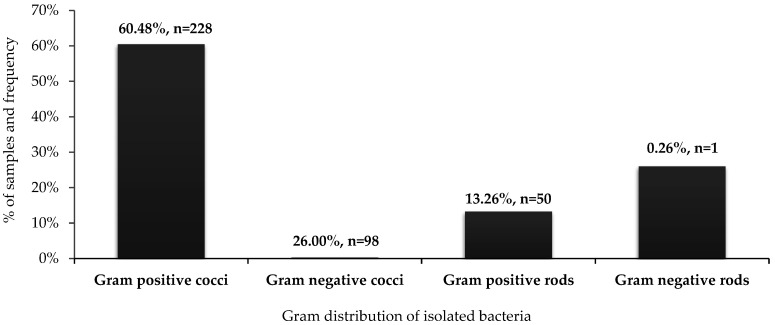
Distribution of identified bacteria according to Gram staining: *n* = 377.

The identified bacteria were divided into four groups according to Gram staining (Figure 2) gram-positive and gram-negative cocci, and gram-positive and gram-negative rods. Gram-positive cocci were the most common—60.48% (*n* = 228), followed by gram-negative rods (26%, *n* = 98), followed by gram-positive rods 13.26% (*n* = 50). Gram-negative cocci occurred very sporadically, and only one species was identified, *Neisseria subflava*, isolated at the DAIC (*n* = 1).

Of the total number of identified bacteria (*n* = 377), 98 strains (26%) belonged to gram-negative rods. Table 1 shows that the most frequently isolated nosocomial gram-negative rod occurring in both departments is *P. aeruginosa* (*n* = 33), followed by *A. baumannii* (*n* = 20) and *E. cloacae* (*n* = 14).

The antibiotic resistance of isolated gram-negative rods varied. As a result, seven resistance phenotypes were identified by Bacmed: AmpC, SHV 1, TEM 1, ESBL, Qnr, MBL, OXA—48.

**Figure 3:**
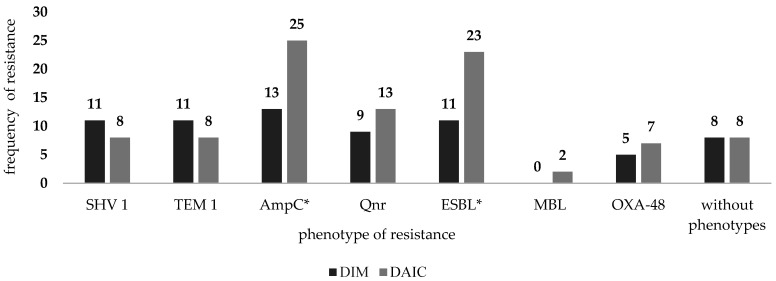
Number of individual phenotypes of resistance in isolated gram-negative nosocomial rods by department (statistically significant in cases AmpC and ESBL, statistically evaluated by chi-square test, *p* = 0.047 for AmpC, and *p* = 0.041 for ESBL, *—significant).

**Figure 4:**
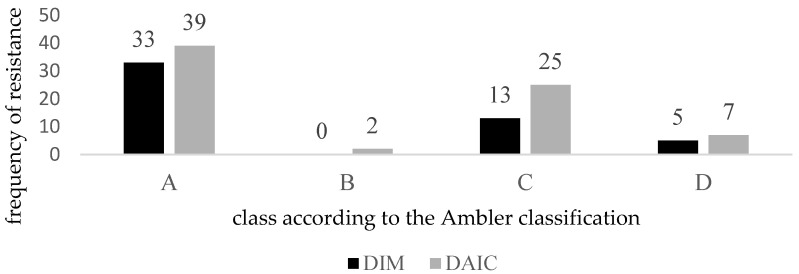
The number of individual types of β-lactamases after division into groups according to Ambler classification [20].

Figure 3 describes the representation of individual phenotypes of resistance in these nosocomial isolates and the number of sensitive nosocomial rods. According to Ambler classification, Figure 4 shows the number of individual types of β-lactamases after division into groups.

The most common resistance phenotype determined in isolated nosocomial rods at both sites was ampicillin resistance. A total of 38 strains had this type of resistance (DIM, *n* = 13; DAIC, *n* = 25). The second most commonly detected type of resistance at the DAIC was ESBL production (*n* = 23), and at the DIM was also ESBL production, along with SHV-1 and TEM-1 (*n* = 11). The third most common type of resistance in both workplaces was resistance to fluoroquinolones—Qnr, (*n* = 22) (DIM, *n* = 9; DAIC, *n* = 13). In 12 cases, the presence of the nosocomially important enzyme OXA-48, capable of hydrolyzing carbapenem antibiotics, was demonstrated. This enzyme was present in five strains isolated from DIM (three strains of *A. baumannii*, two strains of *P. aeruginosa*) and seven strains from DAIC (three strains of *E. cloacae*, three strains of *P. aeruginosa*, and one strain of *K. pneumoniae*). Additionally, nosocomially important carbapenemase MBL (metallo-β-lactamase) occurred in two isolates from DAIC (*P. aeruginosa, K. pneumonaie*). The 16 isolated nosocomial strains were without resistance phenotype (DIM, *n* = 8, DAIC, *n* = 8). Figure 3 shows that strains with ESBL and AmpC resistance phenotypes are statistically more frequent on DAIC (according to the Chi-square test, *p* = 0.047 for AmpC, and *p* = 0.041 for ESBL).

## 4. Discussion

This article describes the identification of bacteria isolated from swabs from inanimate surfaces from two workplaces at the University Hospital, which was carried out using an innovative microbiological method based on mass spectrometry—MALDI-TOF MS. The resistance phenotype was identified in the nosocomial, gram-negative rods using the disk diffusion method and the Bacmed instrument.

Many studies have shown [16,21,22] that inanimate hospital surfaces are a significant factor in spreading important nosocomial pathogens to patients. These include coagulase-negative staphylococci, *Staphylococcus aureus*, MRSA, enterococci, and gram-negative rods, which can be easily isolated from inanimate surfaces near colonized or infected patients. In addition, these microorganisms can survive in a hospital environment for hours and days [23].

There were 102 and 80 swabs taken from the inanimate surfaces of DIM and DAIC, respectively. Overall, 382 pure bacterial cultures were identified, and MALDI-TOF MS successfully identified 377 bacterial strains (98.7%). Yu Zhou et al. [24] reported a 95.5% identification rate of isolated bacterial strains by MALDI. In a study by Guo et al. [25], 97% of all bacteria isolated and cultured were successfully identified using the MALDI method.

Wang et al. [26] reported that the detection rate of bacteria isolated from the inanimate surfaces of routine wards (not providing intensive care) was significantly higher than in the ICU. These were primarily gram-negative bacteria—most often.

*P. aeruginosa*, *E. cloacae*, *A. baumannii*, and *K. pneumoniae*. Our results recorded positive culture from DAIC in 63 of 80 smears (78.8%) and from DIM in 99 of 102 smears (97.1%). A possible cause of this difference is a different local epidemiological situation or a different method of sampling and identification.

Darge et al. [27] stated that out of 130 swabs taken from inanimate hospital surfaces, 115 (88.5%) were culture positive. All swabs (100%) taken from manometers, bedside, and computer tables were contaminated with bacteria. A total of 171 bacterial isolates were identified from culture-positive swabs, where most of the isolates were resistant to ampicillin (as in our results). 

Out of the isolated and identified bacterial strains that occurred on the inanimate surfaces of both workplaces, 98 isolates (26%) belonged to nosocomial gram-negative rods. In a study by Ahmed et al. [28], gram-negative rods isolated from inanimate hospital surfaces accounted for 29.3%. 

The most common nosocomial rod at both sites was *P. aeruginosa*. It formed up to one-third of all isolated nosocomial rods (33.7%, *n* = 33) originating from inanimate surfaces (DIM, *n* = 15; DAIC, *n* = 18). In many publications [29,30], authors stated that *P. aeruginosa* strains are more common in intensive care units (ICU or DAIC). De Abreu et al. [31] stated that *P*. *aeruginosa* strains are relatively common on the inanimate surfaces of common wards. 

In our study, the second most frequently isolated pathogen from inanimate surfaces was *A. baumannii* (20.4%, *n* = 20). It occurred more frequently on inanimate DIM surfaces (*n* = 16). Four strains were identified on the DAIC (*p* = 0.021). The results of Różańska et al. [32] and Weber et al. [33] confirm that isolates of *P. aeruginosa* and *A. baumannii* are the most common gram-negative rods on inanimate hospital surfaces. *A. baumannii* is, according to Rocha et al. [34], one of the most common pathogens causing nosocomial infections. However, according to their results, it was more often isolated from inanimate ICU surfaces, and these were mainly multidrug-resistant strains. Most of the isolates of *A. baumannii* identified in our study came from DIM (*n* = 16). The results of our study correlate with the results of Rocha et al., as all strains of *A. baumannii* (*n* = 4) found on inanimate DAIC surfaces showed resistance to carbapenems (OXA-48). According to Ren et al. [35], clinical isolates of *A. baumannii*, producing carbapenemase OXA-48, are more common in intensive care units, and this colonization leads to the development of infection more frequently. Their occurrence on routine wards is less common and are mostly colonization without clinical manifestations of infection.

We identified 14 strains of *E. cloacae* (14.3%; DIM, *n* = 7; DAIC, *n* = 7). The study by Birru et al. [36] stated that *E. cloacae* isolates are more common on standard wards (less in the ICU).

Muhammad et al. [37] reported that isolates of *K. pneumoniae*, *A. baumannii*, *Escherichia* spp., *C. freundii*, and *P. mirabilis* are the most common on the inanimate surfaces of intensive care units and long-term care facilities. On the other hand, Muhammad et al. managed to isolate only two strains of *P. aeruginosa* from the inanimate surfaces of the mentioned workplaces, which does not correlate with our results. This difference may be, e.g., other local representations of pathogens on inanimate surfaces of the studied workplaces, etc.

Two OXA-48-producing *A. baumannii* strains and 5 ESBL-producing strains were isolated from inanimate DIM surfaces. Zafiri et al. [38] reported that *A. baumannii* ESBL strains are increasingly common on inanimate hospital surfaces—up to 25% of all *Acinetobacter baumannii* isolates obtained from long-term care patients reported ESBL production. In addition, five strains of *A. baumannii* from inanimate DIM surfaces showed resistance to fluoroquinolones.

One-third of *P. aeruginosa* strains (*n* = 5, 33.3%) found on inanimate DIM surfaces showed resistance to carbapenems (OXA-48 production). Slimene et al. [39] reported that out of 82 isolates of gram-negative bacteria obtained from inanimate surfaces of Libya Hospital, eight strains of *A. baumannii* and three strains of *P. aeruginosa* through OXA production showed resistance to carbapenems. Yagoubat et al. [40] reported that out of 99 isolates of gram-negative bacteria from inanimate surfaces, 10.1% (*n* = 10) showed reduced susceptibility to carbapenems (strains *A. baumannii n* = 7, *A. nosocomialis n* = 1, *E. coli n* = 1. and *K. pneumoniae n* = 1). 

The results of Ahmed et al. [28] and Kiros et al. [41] point out that strains of *Klebsiella pneumoniae* are relatively common on inanimate surfaces. However, in our study, only two strains of *K. pneumoniae* (DAIC, *n* = 1; DIM, *n* = 1) were identified from inanimate surfaces. This difference may be related to several factors, e.g., the inanimate surface sampling method, disinfectants used, bacterial cultivability, and other local epidemiological sites, as stated by Otter et al. [42].

This study has several strengths. It was confirmed that the positivity rate of bacterial contamination on inanimate surfaces was high. These findings can strengthen infection control policies and infection control interventions in the hospital. We also consider the practical use of the innovative method of bacterial identification, the foundation of which is mass spectrometry—MALDI-TOF MS, to be the study’s strengths. The main advantage of this method is the length of identification of a pure bacterial culture, which lasts approximately 20 min (from the application of the sample to the target to the successful evaluation). Samples on the target were applied in duplicity to increase the success of bacterial identification.

Our study’s limitation is that the cultivation of bacteria collected from inanimate surfaces took place under aerobic conditions, which does not allow the cultivation of anaerobic bacteria, especially from the genus *Clostridium* spp., which is one of the major causes of nosocomial infections. Another limiting factor was that we did not use molecular techniques (due to financial reasons) to identify bacteria, limiting the observation of species’ diversity and the quantification of the genes involved in these mechanisms. However, culturing on conventional blood agar in some cases does not capture some species of sensitive bacterial strains, e.g., *Haemophilus* spp.

These limitations limited the study results only minimally, as the research focused on the presence of gram-negative rods on inanimate surfaces. The most important genera of nosocomial gram-negative rods grow very well on blood agar under aerobic conditions. In clinical practice, it is more important to know the resistance of nosocomial pathogens to antibiotics than the genetic nature of antibiotic resistance.

## 5. Conclusions

The study aimed to determine the prevalence of gram-negative rods on the inanimate surfaces of two selected workplaces at University Hospital in eastern Slovakia and determine their resistance phenotypes. Knowledge of what pathogens occur on inanimate surfaces near hospitalized patients is of great epidemiological importance. This is mainly because these bacteria often cause nosocomial infections.

Knowledge of the local prevalence and resistance of pathogens colonizing inanimate surfaces helps properly initiate empirical antibiotic treatment for nosocomial infections. Early and well-chosen empirical antibiotic treatment reduces the overall mortality from nosocomial infections. Furthermore, knowledge of the degree of contamination of inanimate surfaces makes it possible to provide various timely interventions to reduce the colonization of inanimate surfaces, e.g., using correctly chosen disinfectants, increasing the quality of hand hygiene, and educating staff about the local hygienic–epidemiological situation, etc.

Based on the results of random bacteriological tests at two different hospital workplaces, this study confirmed that the rate of colonization of inanimate surfaces is high.

The staff’s hands can spread pathogenic bacteria to patients, e.g., by contact of gloves with the infected inanimate surface around the patient, or the gloves themselves may be infected with bacteria. The transmission of bacteria occurs through contaminated diagnostic and therapeutic devices and the laundry. Disinfection of these devices is often inappropriate and can be a vector for the spread of nosocomial bacteria. Proper cleaning and disinfection contribute to the decrease in cross-contamination and, consequently, reduce infections related to contact with surfaces.

Regular monitoring and repeated random sampling of inanimate surfaces in each hospital ward allow a better understanding of the local epidemiological situation. Based on the results of this monitoring, it is possible to develop an intervention plan aimed at combating the spread of nosocomial pathogens, the proper disinfection of inanimate surfaces, and compliance with the principles of hand and environmental hygiene.

## Figures and Tables

**Table 1:** Frequency of selected gram-negative rods at both departments; (*n* = 98 total; *n* = 56, DIM; *n* = 42, DAIC) (statistically evaluated by chi-square test). (NS—not significant, *—significant).

Identified Strain	DIM	DAIC	Overall	*p* (Value)
*Acinetobacter* *baumannii*	16 (28.57%)	4 (9.52%)	20 (20.41%)	*p* = 0.021 *
*Pseudomonas aeruginosa*	15 (26.79%)	18 (42.86)	33 (33.67%)	NS
*Enterobacter cloacae*	7 (12.5%)	7 (16.67%)	14 (14.29%)	NS
*Lecleria adecarboxylata*	5 (8.93%)	1 (2.38%)	6 (6.12%)	NS
*Raoltella planticola*	2 (3.57%)	0 (0%)	2 (2.04%)	NS
*Stenotrophomonas maltophilia*	2 (3.57%)	6 (14.29%)	8 (8.16%)	NS
*Citrobacter braakii*	1 (1.79%)	2 (4.76%)	3 (3.06%)	NS
*Citrobacter freundii*	1 (1.79%)	0 (0%)	1 (1.02%)	NS
*Escherichia hermannii*	1 (1.79%)	0 (0%)	1 (1.02%)	NS
*Escherichia vulneris*	1 (1.79%)	0 (0%)	1 (1.02%)	NS
*Klebsiella pneumoniae*	1 (1.79%)	1 (2.38%)	2 (2.04%)	NS
*Pantoea calida*	1 (1.79%)	0 (0%)	1 (1.02%)	NS
*Proteus mirabillis*	1 (1.79%)	0 (0%)	1 (1.02%)	NS
*Proteus vulgaris*	1 (1.79%)	0 (0%)	1 (1.02%)	NS
*Providencia rettgeri*	1 (1.79%)	0 (0%)	1 (1.02%)	NS
*Enterobacter asburiae*	0 (0%)	1 (2.38%)	1 (1.02%)	NS
*Pantoea aglomerans*	0 (0%)	1 (2.38%)	1 (1.02%)	NS
*Serratia marcescens*	0 (0%)	1 (2.38%)	1 (1.02%)	NS
Total	56	42	98	

## Data Availability

Not applicable.
